# Preparation of Copper Nanoplates in Aqueous Phase and Electrochemical Detection of Dopamine

**DOI:** 10.3390/life12070999

**Published:** 2022-07-05

**Authors:** Lijian Xu, Sijia Tang, Ling Zhang, Jingjing Du, Jianxiong Xu, Na Li, Zengmin Tang

**Affiliations:** 1 Hunan Key Laboratory of Biomedical Nanomaterials and Devices, College of Life Sciences and Chemistry, Hunan University of Technology, Zhuzhou 412007, China; xlj235@163.com (L.X.); t3465188455@163.com (S.T.); lingzhang645@126.com (L.Z.); xujianxiong8411@163.com (J.X.); 2 College of Packaging and Materials Engineering, Hunan University of Technology, Zhuzhou 412007, China; djj19820923@126.com; 3 Hunan Key Laboratory of Electrochemical Green Metallurgy Technology, College of Materials and Advanced Manufacturing, Hunan University of Technology, Zhuzhou 412007, China; lina@hut.edu.cn

**Keywords:** copper nanoplate, Ag nanoparticles, synthesis, modified glass carbon electrode, dopamine

## Abstract

Compared with gold and silver, cheap copper has attracted more attention and can potentially be applied in non-enzymatic electrochemical sensors due to its excellent conductivity and catalytic activity. In this paper, copper nanoplates were rapidly synthesized using copper bromide as the copper precursor, polyethyleneimine as the stabilizer, and ascorbic acid as a reducing agent in the presence of silver nanoparticles at a reaction temperature of 90 °C. The Cu nanoplates with an average side length of 10.97 ± 3.45 μm were obtained after a short reaction time of 2 h, demonstrating the promoting effect of an appropriate amount of silver nanoparticle on the synthesis of Cu nanoplates. Then, the electrochemical dopamine sensor was constructed by modifying a glass carbon electrode (GCE) with the Cu nanoplates. The results obtained from the test of cyclic voltammetry and chronoamperometry indicated that the Cu-GCE showed a significant electrochemical response for the measurement of dopamine. The oxidation peak current increased linearly with the concentration of dopamine in the range of 200 µmol/L to 2.21 mmol/L, and the corresponding detection limit was calculated to be 62.4 μmol/L (S/N = 3). Furthermore, the anti-interference test showed that the dopamine sensor was not affected by a high concentration of ascorbic acid, glucose, uric acid, etc. Therefore, the constructed Cu-GCE with good selectivity, sensitivity, and stability possesses a high application value in the detection of dopamine.

## 1. Introduction

Among the numerous metallic nanomaterials such as silver and gold, copper (Cu) is one thousand times more abundant than silver in the earth’s crust, it is one hundred times cheaper than silver, and it is six thousand times cheaper than gold. In recent decades, Cu has received considerable attention due to its inexpensiveness, high abundance, good electrical conductivity, and wide application in various fields, such as catalysis (4-nitrophenol reduction, electrocatalytic oxygen reduction, and electrocatalytic carbon dioxide reduction), as antibacterial agents, in electronic devices (electronic ink and flexible transparent electrodes), and in electrochemical sensors (hydrogen peroxide, glucose, and dopamine) [1,2,3,4,5,6,7]. The performance of nanoparticles in various applications greatly depends on their morphologies and surface chemistries [8,9,10,11,12]. Recently, the various copper nanoparticles in shapes such as a wire, rod, cube, polyhedron, disk, sheet, and triangle/hexagonal plates have been obtained by developing the “bottom-up” approach [13,14,15,16].

During the synthesis process, especially for anisotropic two-dimensional (2D) Cu nanoplates, the fabrication conditions are time-consuming, involve the use of toxic substances, require high temperatures, etc., and are thus not conducive to the practical applications of products. In 2009, I. Pastoriza-Santos synthesized Cu nanoplates in the solvent of N, N-dimethylformamide, which was conducted at 60 °C and only took 3–4 min due to the use hydrazine as a strong reducing agent [17]. With its toxicity and corrosiveness, hydrazine may be harmful to the environment and is incapable of meeting the requirements of green chemistry principles. Moreover, the organic solvent of N, N-dimethylformamide increases the burden of post-processing steps of Cu nanoplates. Subsequently, S. Xu et al. reported a hydrothermal process to synthesize the Cu nanoplates by using ascorbic acid worked as a green reducing agent and found that the halide element played an important role in regulating the morphology of Cu nanoparticles [18]. In the presence of an insoluble substance (cuprous chloride) and a mild reducing agent, a higher energy input (high temperature of 120 °C) was required to initiate the synthetic reaction of the Cu nanoplates. On this basis, J. Lee et al. used CuCl_2_ as a precursor, glucose as a reducing agent, and hexadecylamine as a stabilizer to synthesize Cu nanoplates with the assistance of a small amount of iodine [19]. This process reduced its temperature, but the reaction time was extended to 12 h. In addition, the insoluble hexadecylamine led to the formation of an emulsion system that could easily coat the surface of products, which eventually needed to be cleaned by chloroform. For a more convenient synthesis of Cu nanoplates, Z. Tang selected soluble polyethyleneimine (BPEI) as an efficient stabilizer, which contains a large number of amine groups [20]. In the process, the synthesis and growth of Cu nanoplates required at least 10 h in the presence of Br^−^ ion and polyvinyl-pyrrolidone; thus, it is a process that can be improved further.

The achievement of a concise synthetic route is also a pursued goal in the research of nanoparticles. This paper further improved the original synthetic method for producing Cu nanoplates, which was synthesized by using water as the solvent, CuBr_2_ as the precursor, ascorbic acid (AA) as a reducing agent, and BPEI as a stabilizer. In this synthetic process, a small amount of Ag nanoparticles was added and seemed to act as a catalyst to accelerate the formation of Cu nanoplates. At 90 °C, the reaction time was reduced to 2 h. Moreover, the synthesized Cu nanoplates were used to construct a modified electrode for the electrochemical detection of dopamine.

## 2. Materials and Methods

### 2.1. Materials

Cupric bromide (CuBr_2_, 99%), L-ascorbic acid (C_6_H_8_O_6_, AA 99%), dopamine hydrochloride (C_8_H_11_NO_2_·HCl, 98%), disodium hydrogen phosphate (Na_2_HPO_4_, ≥99%), Nafion solution (Perfluorosulfonic acid-PTFE copolymer, 5% in a mixture of lower aliphatic alcohols and water), sodium dihydrogen phosphate (NaH_2_PO_4_, 99%), hydrogen peroxide (H_2_O_2_, 30 wt% solutions in water), uric acid (C_5_H_4_N_4_O_3_, 99%), and polyethyleneimine (BPEI, 50 wt% solutions in water, MW: 60,000) were obtained from InnoChem and used without purification. Water was purified by ion exchange (D.I. water).

### 2.2. Synthesis of Cu Nanoplates

During the synthetic process, 0.1 mmol CuBr_2_ and 20 mg BPEI were dissolved in 7 mL of deionized water to obtain a blue transparent solution in a 90 °C water bath. Then, the 40 μL of pre-prepared Ag nanoparticle suspension with a concentration of 3 mmol/L was added to the above blue solution, followed by the addition of 3 mL of ascorbic acid solution (0.6 mol/L). Stirring of the mixed solution was maintained during the overall process. After 2 h, a brownish-red reaction suspension containing Cu nanoplates was obtained and cooled down to room temperature, and then the Cu nanoplates were collected and washed.

Additionally, the Ag nanoparticles used for the synthesis of Cu nanoplates were prepared by using AgNO_3_ as the precursor, BPEI as a stabilizer, and ascorbic acid as a reducing agent in an aqueous solution. In detail, first, 0.1 mL AgNO_3_ solution (1 mol/L) was added to 7 mL of water in the presence of BPEI (10 mg). The above mixture was stirred evenly for 30 s on a magnetic stirrer at the temperature of 80 °C. Upon the addition of 1 mL of ascorbic acid solution (0.6 mol/L), a milky suspension was immediately formed and was stirred for 10 min to complete the reaction. Finally, an Ag suspension with a concentration of 0.0123 mol/L was obtained, which was further prediluted to 3 mmol/L employed for the synthesis of Cu nanoplates.

### 2.3. Electrochemical Detection of Dopamine

Electrochemical measurements including electrochemical impedance spectroscopy, cyclic voltammetry, and chronoamperometry were performed in PBS solution (0.2 mol/L, pH = 7) on a CHI660E workstation (Chenhua, Shanghai, China) using a universal three-electrode system (platinum plate functioned as the counter electrode, untreated or modified GCE was used as the working electrode, and saturated calomel electrode functioned as the reference electrode). The 7 μL of Cu nanoplate suspension with a concentration of 5.3 mg/mL was dropped on the polished GCE and dried. Then, 5 μL of 0.01% dimethylformamide-Nafion solution was dropped on the pretreated GCE and dried in a desiccator to finally obtain Cu-GCE.

### 2.4. Characterizations

The products were analyzed by scanning electron microscopy (SEM, Zeiss Gemini 300, Oberkochen, Germany) to observe their morphologies. The crystal structure and elements of the products were analyzed by X-ray diffractometer (XRD, Bruker D8, Karlsruhe, Germany) and X-ray photoelectron spectroscopy (XPS, Thermo Kalpha, Waltham, MA, USA). The performance of the modified electrode was detected by an electrochemical workstation (CHI660E, Shanghai, China). The concentration of Cu nanoplates was measured by Inductively Coupled Plasma–Mass Spectrometry (ICP-MS, PerkinElmer ICP 2100, Waltham, MA, USA).

## 3. Results and Discussion

### 3.1. Characterization and Analysis of Copper Nanoplate

**Scheme 1:**
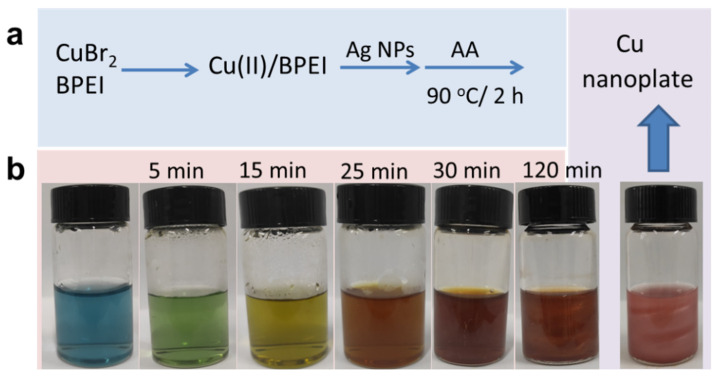
Schematic diagram for the synthesis of Cu nanoplates (**a**) and photograph (**b**) in fabricated process.

In an aqueous solution at 90 °C, the Cu nanoplates were fabricated by the reaction of CuBr_2_ with ascorbic acid in the presence of BPEI and a small amount of Ag nanoparticles (30 μL) as shown in Scheme 1. In the synthetic process, the color of the reaction solution changed from blue to green (after 5 min), to light yellow (after 15 min), and finally to brown (after 25 min), which reveals the transformation of bivalent Cu^2+^ to a zero-valent Cu atom. After 30 min, the color deepened into brownish red and a few visible particles appeared in the solution. The reaction solution reacted for a further 90 min to ensure a complete reaction.

**Figure 1:**
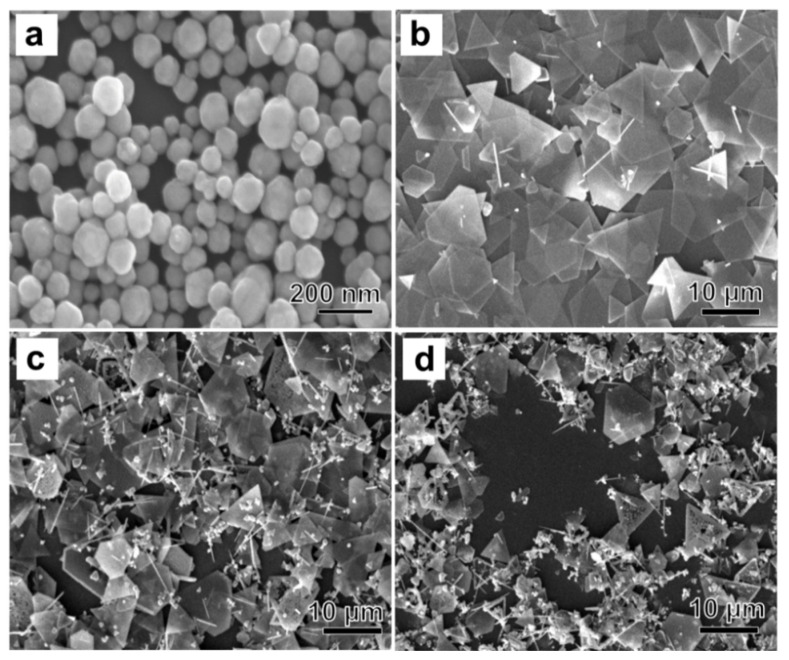
(**a**) SEM images of Ag nanoparticles, (**b**–**d**) SEM images of samples obtained at different addition amounts of Ag nanoparticle suspension: (**b**) 30 μL, (**c**) 60 μL, and (**d**) 100 μL.

The shapes of the products were measured by SEM. The SEM image in Figure 1a shows the quasi-spheric Ag nanoparticles with a mean size of 81.5 ± 5.68 nm. In the presence of Ag nanoparticles, the synthesis of Cu nanoplates is promoted. It can be observed from Figure 1b that the products are mainly composed of triangular and hexagonal plates with an average side length of 10.97 ± 3.45 μm. As the amount of Ag nanoparticle suspension is increased to 60 μL and 100 μL, the products are mixed, with large amounts of wire, plate, and particle shapes, as shown in Figure 1c,d. Moreover, the nanoplates become smaller; the average side length decreased to 7.66 ± 2.82 μm and 4.85 ± 2.14 μm. Compared with the previous method, the presence of Ag nanoparticles with a moderate amount can effectively promote the nucleation and growth of Cu nanoplates [21]. The reaction time required to synthesize Cu nanoplates was shortened at least five times. An excessive amount of Ag nanoplates would lead to a fast but uncontrollable nucleation rate, which is not favorable for the growth of anisotropic Cu nanoplates.

**Figure 2:**
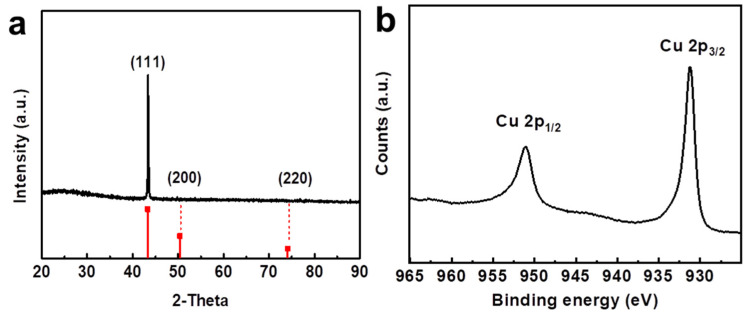
(**a**) XRD pattern of Cu nanoplates and (**b**) XPS of Cu element in Cu nanoplates.

The crystal structure and element valence state were analyzed by XRD and XPS. The results are shown in Figure 2. Figure 2a shows a typical powder XRD pattern of the prepared Cu nanoplates; it is consistent with the standard card number of copper JCPDS #04-0836 [22]. In the XRD pattern, a strong absorption peak at 2θ = 43.4 corresponds to the copper <111> crystal plane, and the other two peaks do not appear due to the weak peak intensity, which illustrates that the exposure facet of the Cu nanoplates is (111) facet [23]. In addition, the diffraction peaks of impurities such as Cu_2_O and CuO were not detected, which shows that the synthesized copper nanoplates have high purity. The XPS spectrum of Cu in Figure 2b demonstrates two peaks at 932.3 and 952.2 eV, assigning core levels to Cu 2p3/2 and 2p1/2, which further demonstrates the existence of metallic Cu instead of the oxidation state of Cu [24].

### 3.2. Electrochemical Behavior of Cu-GCE

**Figure 3:**
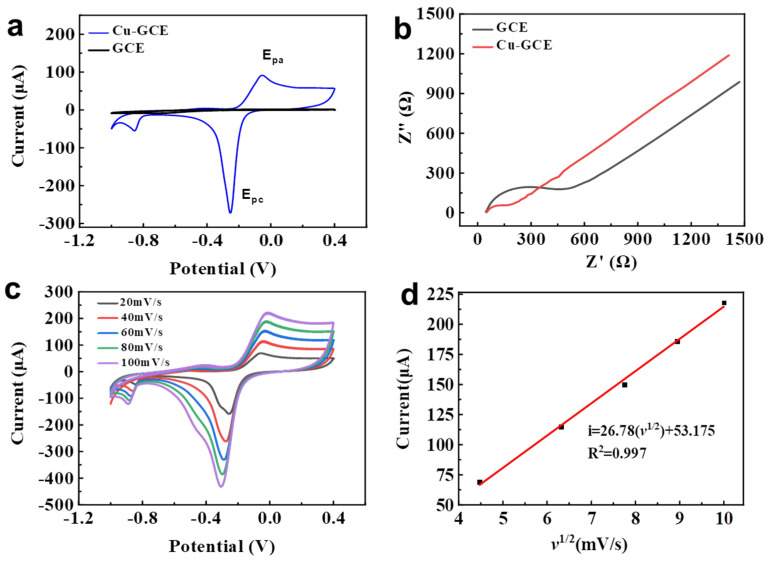
(**a**) Cyclic voltammograms of the bare GCE and Cu-GCE in PBS solution at a scanning rate of 40 mV/s, (**b**) the EIS of the bare GCE and Cu-GCE, (**c**) cyclic voltammograms of Cu-GCE in PBS solution at different scanning rates (**d**), and the fitting line of peak current at E_pa_ to the square root of the scanning rate.

Figure 3a demonstrates the cyclic voltammograms of bare and modified electrodes in the 0.2 mol/L PBS solution at a scan rate of 40 mV/s. It reveals that bare GCE has no obvious response in the potential range from −1.0 V to +0.4 V. Instead, Cu-GCE displays a pair of well-defined redox peaks with a strong response. The anodic peak potential (E_pa_) of −0.056 V and the cathodic peak potential (E_pc_) of −0.253 V are attributed to the oxidation of Cu and the reduction of Cu^2+^ ions [25]. This illustrates that the GCE modified by Cu nanoplates displays better performance than bare GCE. Figure 3b shows the Nyquist plots consisting of a semicircle part and a linear potion, which indicates that Cu-GCE had a relatively smaller R_ct_ than that of bare GCE, which further confirms that an enhanced charge transfer rate of Cu-GCE occurred due to the Cu nanoplates. Figure 3c shows the cyclic voltammograms of Cu-GCE at a series of scanning rates. Since the scanning rates varied from 20 mV/s to 100 mV/s, the peak current corresponding to the oxidation peak has a good linear relationship with the scanning rate. In Figure 3d, the linear equation can be expressed as *i* = 48.6067 + 54.8266*v*^1/2^, and the correlation coefficient (R^2^) is 0.9987, which indicates that the electrochemical behavior of the copper nanosheets on the modified electrode is controlled by surface adsorption.

### 3.3. Electrochemical Detection of Dopamine in Cu-GCE

**Figure 4:**
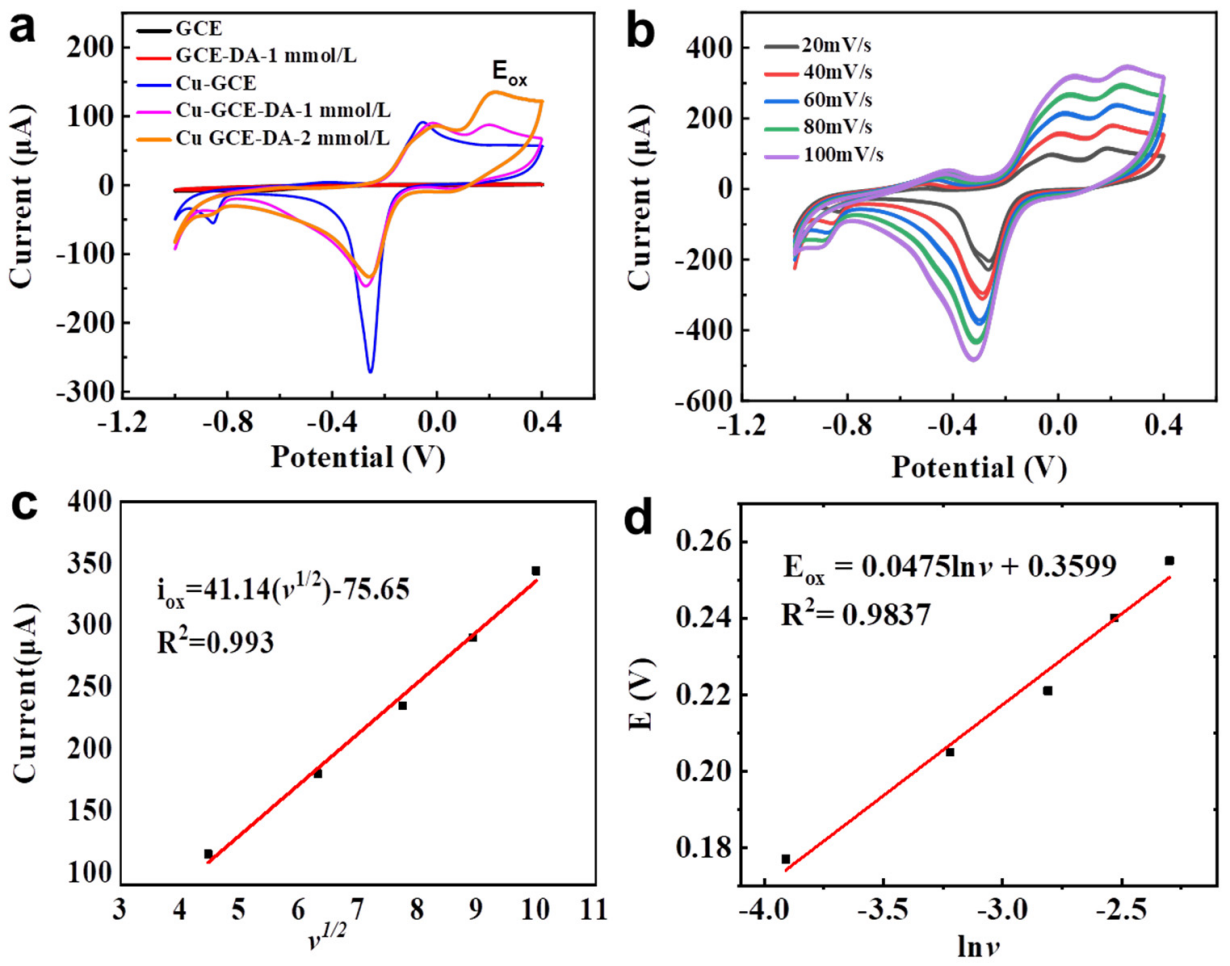
(**a**) Cyclic voltammograms of the bare GCE and Cu-GCE in the presence of dopamine at a scanning rate of 40 mV/s, (**b**) cyclic voltammograms of Cu-GCE in PBS solution with dopamine concentration of 1 mmol/L at different scanning rates, (**c**) the fitting line of peak current at E_ox_ to the square root of the scanning rate (**d**), and plots of E_ox_ to Napierian logarithm of scan rate.

In a phosphate buffer solution of pH = 7, the bare GCE and Cu-GCE were evaluated for their detection of dopamine. The results are shown in Figure 4a. In the presence of dopamine with a concentration of 1 mmol/L, the cyclic voltammogram of Cu-GCE indicates that a new oxidation peak (E_ox_) caused by the oxidation of dopamine appears at the potential of 0.2 V and the corresponding current peak is 86.5 μA. When the concentration of dopamine was continuously increased to 2 mmol/L, the response current of the Cu-GCE also increased to 136.2 μA accordingly. However, the bare GCE only has a very weak current response, which can be negligible in contrast. Therefore, the results indicate that the constructed Cu-GCE can respond to both the presence and the change of dopamine concentration. Figure 4b demonstrates that the currents at the potential of E_ox_ also increase with the increase of scan rate from 20 mV/s to 100 mV/s. As shown in Figure 4c, the current of E_ox_ is quite linear to the root of the scanning rate, and the linear equation is expressed as *i*_ox_ = 41.14*v*^1/2^ − 75.65 (R^2^ = 0.993), which reveals that the electrochemical oxidation of dopamine on the Cu-GCE is controlled by an adsorption-controlled process. On further analysis, the E_ox_ of oxidation peak shifts positively with the increase of the scan rates, and the result is presented in Figure 4d. The E_ox_ is linear to the Napierian logarithm of scan rate (ln*v*), which can be described by the equation E_ox_ = 0.0475ln*v* + 0.3599 (R^2^ = 0.9837). According to equation [26,27]:

E = E° + (RT/αnF) ln(RT*k*°/αnF) + (RT/αnF) ln*v*

In the above equation, the T is Kelvin temperature (K), R is the ideal gas constant (8.314 J/(mol·K)), *k*° is heterogeneous electron transfer rate, E° is the formal potential (V), F is Faraday constant (96,480 C/mol), and α is generally 0.5 for a quasi-reversible process. Then, *n* is calculated to be around 1. Therefore, the oxidation of dopamine is a one electron transferred quasi-reversible process [28].

**Figure 5:**
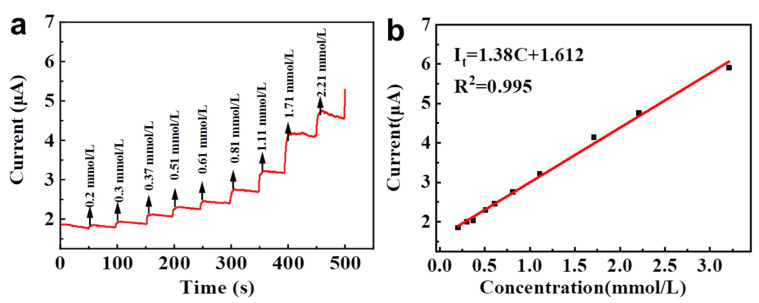
(**a**) The chronoamperometric response of the Cu-GCE with the increase of added dopamine concentration in PBS solution at an applied potential of +0.2 V and (**b**) calibration curves between response peak currents and the concentration of dopamine.

To systematically analyze the response behavior of the modified electrode to dopamine, the chronoamperometric response of the Cu-GCE was evaluated through the successive injection of DA with concentrations into the 0.2 M PBS and the voltage was fixed at 0.2 V. As shown in Figure 5a, after the addition of dopamine, the response current of the Cu-GCE nearly reaches the steady-state value within <3 s, which is indicative of a rapid response. In the concentration range of 0.2−2.21 mmol/L of dopamine, the current value (I_t_) is linearly positively correlated with the concentration of dopamine (Figure 5b). The linear equation is I_t_ = 1.38C + 1.612 with a correlation coefficient (R^2^) of 0.995. The corresponding detection limit was calculated to be 62.4 μmol/L (S/N = 3), which has a competitive performance towards the detection of dopamine compared with the other materials as shown in Table 1.

### 3.4. Selectivity and Stability of Cu-GCE in Electrochemical Detection of Dopamine

**Figure 6:**
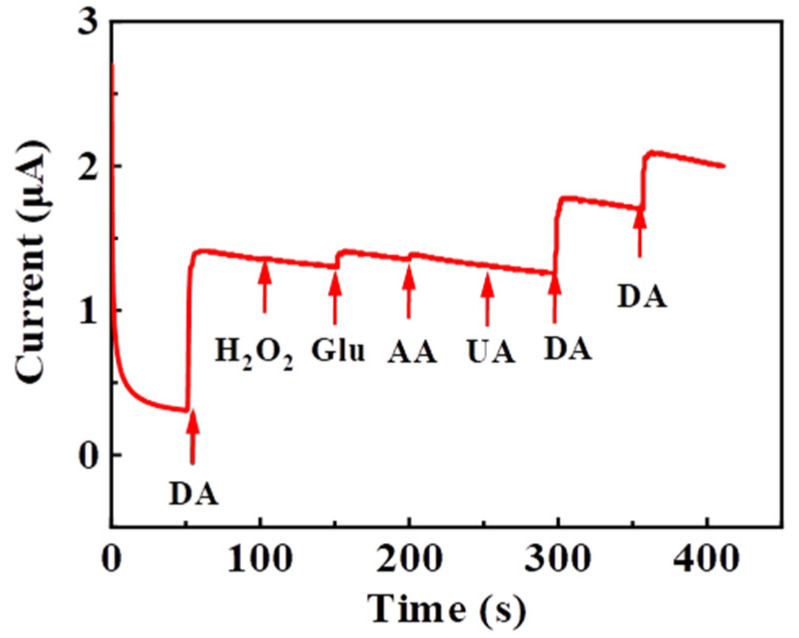
Selectivity of Cu-GCE in electrochemical detection of dopamine.

To evaluate the selectivity of the Cu-GCE for dopamine, the effects of common interfering substances (such as AA, UA, H_2_O_2_, and glucose) on the electrocatalytic performance were determined using chronoamperometry at the voltage of +0.2 V. Figure 6 shows the current change as the addition of dopamine (1 mmol/L) and the interferents (3 mmol/L) to PBS solution, which indicates that the anode current response produced by dopamine is much higher than that of the mentioned interferents. Therefore, these results indicate that the Cu-GCE sensor possesses good selectivity and sensitivity for the detection of DA even in the presence of the interfering substance.

**Figure 7:**
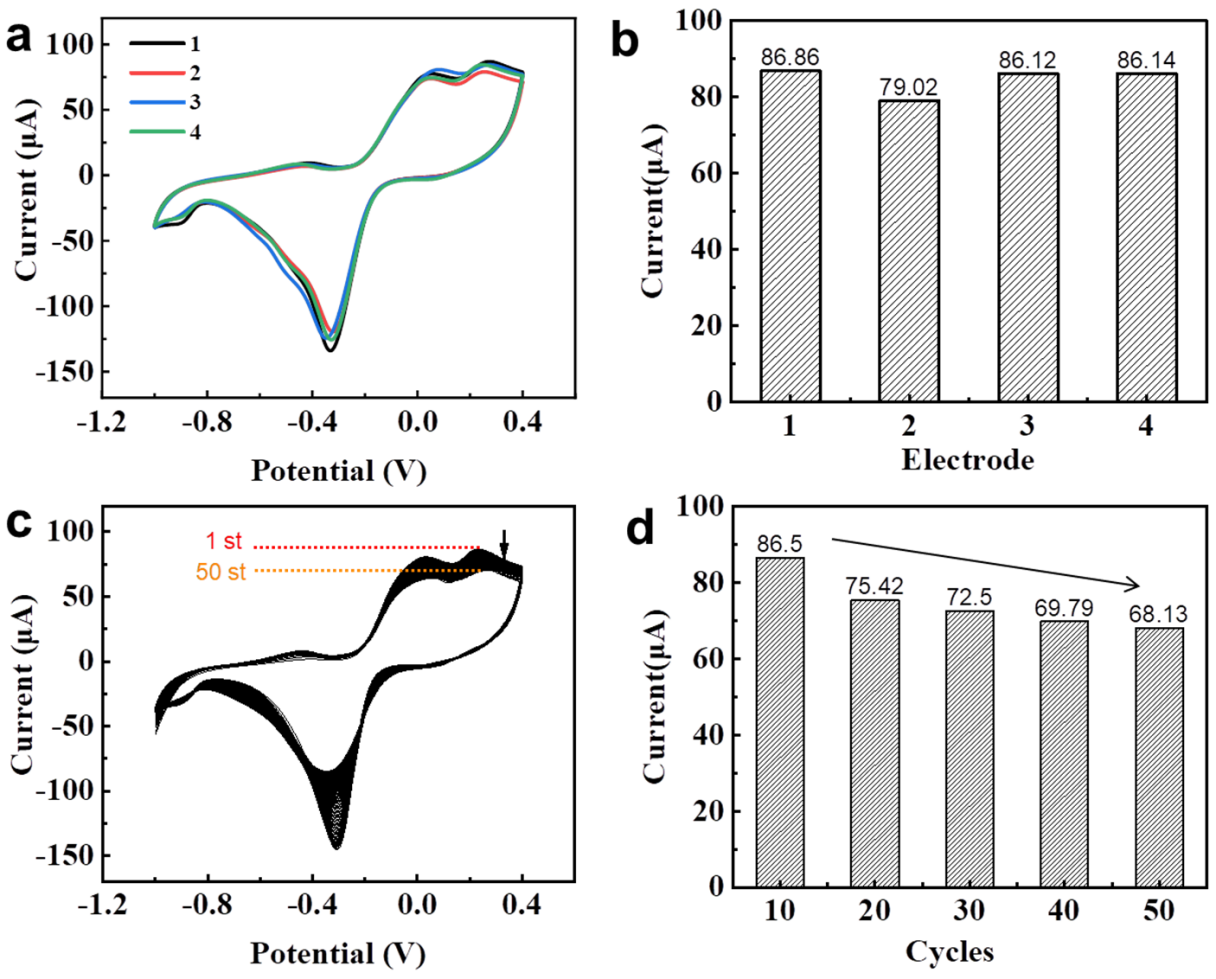
CV (**a**) and *i*_ox_ (**b**) of four independent Cu-GCE and 50 repeated CV (**c**) and *i*_ox_ (**d**) of Cu-GCE in 1 mmol/L at a scanning rate of 40 mV/s.

The accuracy and practicability of the proposed method were verified by analyzing the repeatability and stability of Cu-GCE electrodes. In the reproducibility experiments, four parallel tests were conducted by four independent Cu-GCE in the 1 mmol/L dopamine solution. The resulting relative standard deviation was around 4.36% (Figure 7a,b), which indicates the excellent reproducibility of the prepared Cu-GCE for the detection of DA. Moreover, the stability of Cu-GCE towards dopamine (1.0 mmol/L) detection was tested for 50 multip CV cycles (Figure 7c,d). There was a reduction of 21.2% in the current response, which indicates that the stability of Cu-GCE must be further improved.

## 4. Conclusions

Herein, the synthesis of Cu nanoplates was improved with the assistance of Ag nanoparticles. A suitable amount of Ag nanoparticles can promote the growth of Cu nanoplates and effectively shorten the time required for the synthesis of Cu nanoplates. The Cu nanoplates with the size of 10.97 ± 3.45 μm can be collected after 2 h for reaction. In the electrochemical detection of dopamine, the constructed Cu-GCE showed a good performance. The oxidation peak current possessed a good linear relationship, with the concentration of dopamine in the range of 200 µM to 2.21 mM with a detection limit of 62.4 μmol/L (S/N = 3). Further experiments, including the anti-interference test and reproducibility test, revealed that the Cu-GCE showed high selectivity and good reproducibility, thus contributing to the development of a convenient strategy for the detection of dopamine. The stability of Cu-GCE may be further improved by the modification of Cu nanoplates.

## Figures and Tables

**Table 1:** Comparison of electrochemical detection of dopamine.

Electrode	LOD	Linear Range	Ref.
Au-graphene	1.86 mmol/L	5–1000 mmol/L	[28]
3D-GN@WO_3_ nanowire	238 μmol/L	10–150 mmol/L	[29]
Graphene	2.64 mmol/L	4–100 mmol/L	[30]
ZnO/MWNTs/GCE	3 μmol/L	3–200 μmol/L	[31]
TiO_2_/Graphene	2 μmol/L	5–200 μmol/L	[32]
Cu_3_(BTC)_2_/GCE	15 μmol/L	0.5–100 μmol/L	[33]
MWCNT/(Cu(sal-ala)bpy)/GCE	2.34 μmol/L	0.1–300 μmol/L	[34]
Cu_2_O-RGO/GCE	6 nmol/L	1–100 μmol/L	[6]
CuO nanowires/GCE	0.1 μmol/L	0.1–105 μmol/L	[35]
Cu nanoplate/GCE	62.4 μmol/L	0.2–2.21 mmol/L	This work

## Data Availability

Not applicable.
